# Electrostatic Aspect of the Proton Reactivity in Concentrated Electrolyte Solutions

**DOI:** 10.1021/acs.jpclett.4c02923

**Published:** 2024-12-03

**Authors:** Alicia van Hees, Chao Zhang

**Affiliations:** Department of Chemistry-Ångström Laboratory, Uppsala University, Lägerhyddsvägen 1, P.O. Box 538, 75121 Uppsala, Sweden

## Abstract

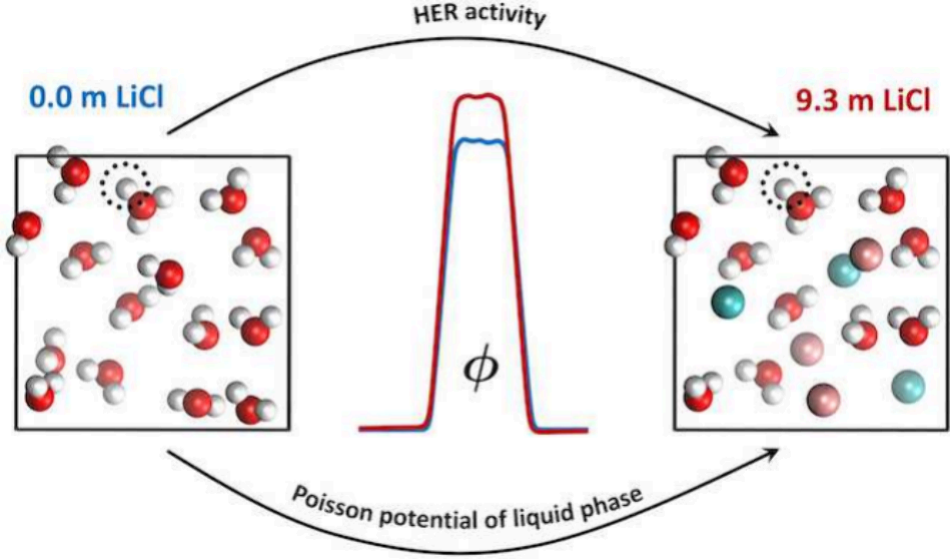

Water-in-salt electrolytes with a surprisingly large electrochemical stability window of ≤3 V have revived interest in aqueous electrolytes for rechargeable lithium-ion batteries. However, recent reports of acidic pH measured in concentrated electrolyte solutions appear to be in contradiction with the suppressed activity of the hydrogen evolution reaction (HER). Therefore, the fundamental thermodynamics of proton reactivity in concentrated electrolyte solutions remains elusive. In this work, we have used density functional theory-based molecular dynamics (MD) simulations and the proton insertion method to investigate how the HER potential shifts in concentrated LiCl solutions under both acidic and alkaline conditions. Our results show that the intrinsic HER activity increases significantly with the salt concentration under acidic conditions but remains relatively constant under alkaline conditions. Moreover, by leverage over finite-field MD simulations, it is found that a determining factor for the HER activity is the Poisson potential of the liquid phase, which increases in concentrated electrolyte solutions with comparable values from both density functional theory and point-charge models.

The reactivity of protons in highly concentrated electrolytes has recently attracted a great deal of interest due to the success of water-in-salt electrolytes (WiSEs) in allowing much wider electrochemical stability windows (ESWs) for rechargeable aqueous batteries.^1,2^ Compared to the general agreement on the enlarged ESW at the positive electrode because of anion accumulation,^3^ the relationship between the hydrogen evolution reaction (HER) activity and the salt concentration at the negative electrode remains elusive.

Different factors contribute to the conundrum of the HER activity in concentrated electrolyte solutions. It was found that the onset potential of HER does not depend on the salt concentration for LiCl and LiNO_3_ electrolytes.^4^ On the contrary, the distinction between free water and bound water in LiTFSI electrolyte solutions turns out to play a crucial role in the HER activity and the subsequent chemical decomposition of TFSI anions.^5^ In addition to the anion type, impurities in LiTFSI electrolytes have been recently reported to have a strong inhibitory effect on the HER.^6^ In addition, the same set of experiments also showed that the exchange current density for the HER is reduced by a factor of 32 with an increase in salt concentration from 1 to 20 *m* (molality), which highlights the importance of the kinetic factor in the reported onset potentials.

Among the reported investigations, only a handful of studies were carried out to understand the equilibrium potential of the HER in concentrated electrolyte solutions. It has been suggested that significant parts of the concentration-dependent shift in the open circuit voltage (OCV) for both LiCl (∼400 mV) and LiTFSI (∼250 mV) electrolytes as measured by the ion selective electrode originate from the changes in liquid junction potentials.^7^ Despite that, this study suggested that protons are indeed created with an increase in the salt concentration. This agrees with a previous report showing that the pH of LiTFSI solutions decreases from 6.5 to 2.4 with an increase in the salt concentration within the same range from 1 to 20 *m*.^8^ Similarly, the authors of ref (9) found that the effective concentration of protons increases at high LiCl concentrations. On the contrary, a recent computational study with density functional theory-based molecular dynamics (DFTMD) simulations^10^ showed that the reduction potential of the TFSI anion is insensitive to the salt concentration, while the water reduction potential decreases significantly, which indicates a reduced proton reactivity. Therefore, it is unclear whether the thermodynamic reversible potential for the HER would increase or decrease with an increase in salt concentration.

A parallel but highly relevant development pointed out the role of the “liquid Madelung potential” in single-ion reactivity. It has been recently shown that the reduction potential of Li^+^ ions deviates significantly from the Nernstian behavior in highly concentrated LiFSI/ethylene carbonate solutions and correlates very well with the liquid Madelung potential,^11^ i.e., the Coulombic potential experienced by the cation due to the surrounding ions in the liquid phase. The computed liquid Madelung potential using classical force fields indicates an increment of ∼400 mV with an increase in the salt concentration from 0 to 8 *m*, which is not too different from the shift in the OCV mentioned previously for aqueous electrolyte solutions. Given that both ethylene carbonate and water are polar liquids with high dielectric constants, it would be very interesting to determine the role of the liquid Madelung potential in aqueous electrolytes.

With these questions in mind, we studied how the equilibrium potential of the HER changes with the salt concentration in LiCl solutions under both acidic and basic conditions with DFTMD simulations. LiCl does not enjoy the same water-in-salt stardom as LiTFSI; nevertheless, it is a model system used in experiments to study concentrated electrolyte solutions. Moreover, the simple LiCl salt provides a convenient starting point for establishing our computational protocols. We showed that the intrinsic HER activity increases significantly with salt concentration under acidic conditions but remains relatively constant under alkaline conditions. This reconciles the seemingly contradicting results from previous experimental and computational studies and emphasizes the role of the water dissociation reaction in HER activity. Moreover, by exploring finite-field MD simulations of bulk electrolyte solutions, we were able to single out the important contribution of the Poisson potential of the liquid phase to the shift of HER potentials in concentrated electrolyte solutions. In particular, it is found that the shifts in the Poisson potential from the neat liquid water to the 9.3 *m* LiCl electrolyte solution turn out to be comparable (∼550 mV in the DFT model and ∼170 mV in the point-charge model) despite the difference in absolute value that is orders of magnitude. This highlights the crucial aspect of a liquid electrostatic environment for determining the proton reactivity in concentrated electrolyte solutions.

First, we will set the stage and introduce the thermodynamics of proton reactivity in concentrated electrolyte solutions and point out different factors that influence the equilibrium potential of the HER.

The HER under acidic conditions is expressed as follows:
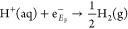
1

The corresponding equilibrium potential for the HER can be determined by following the thermodynamic cycle, as illustrated in Figure 1. The formation energy of H^+^ in the gas phase (Δ_f_*G*_H^+^(g)_^°^) is 15.81 eV, and the standard proton work function (*W*_H^+^_) is 11.36 eV.^13^ That is why the absolute potential of the standard hydrogen electrode (SHE) is *E*_H^+^/H_2__^°,abs^ = −*ΔG*_SHE_^0^/*F* = 4.45 V, where *F* is the Faraday constant.

**Figure 1:**
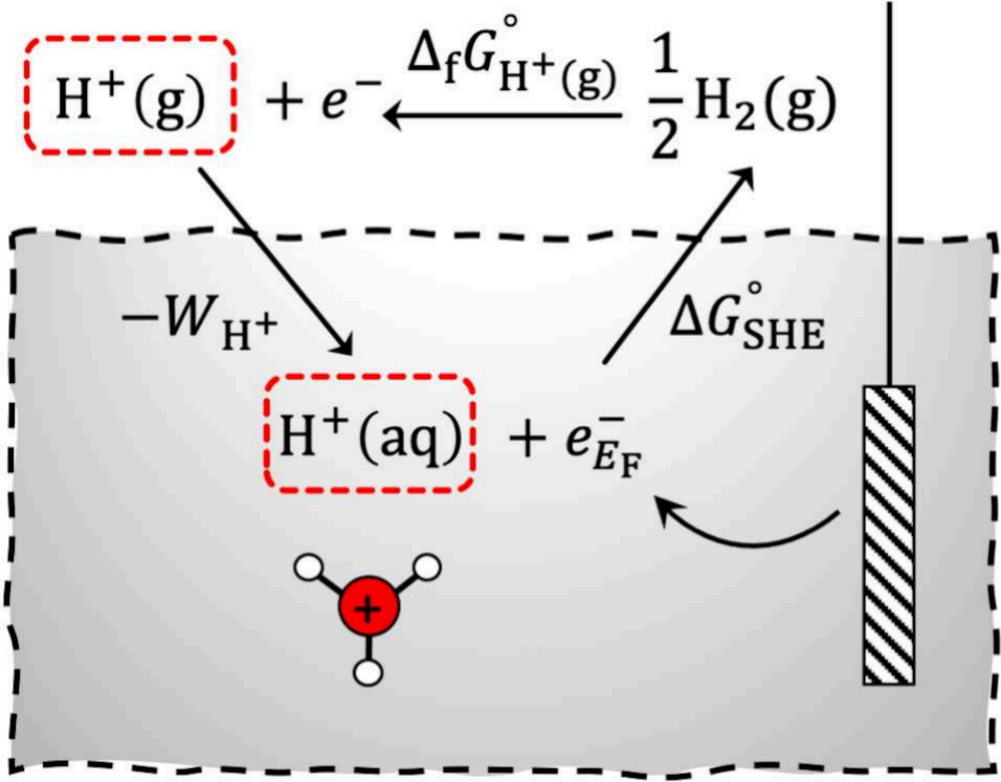
Thermodynamic cycle for determining the standard hydrogen electrode potential under acidic conditions. Adapted from ref (12) under a CC-BY license (copyright 2024 Zhang et al.).

Δ_f_*G*_H^+^(g)_^°^ is a constant in the gas phase, which is of course independent of the nature of salt and its concentration in electrolyte solutions, but this is not the case for proton work function *W*_H^+^_. Therefore, the resulting HER potential under acidic conditions in a concentrated electrolyte (salt) solution of *x* molality and on the SHE scale is
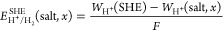
2

For the HER under alkaline conditions, the water dissociation reaction is involved:
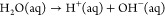
3with equilibrium constant *K*_wd_, the acidity constant p*K*_w_ = −log(*K*_wd_) and the reaction free energy Δ_wd_*G*° = 2.30*RT* × p*K*_w_, where *R* is the gas constant and *T* is the temperature.

Combining eqs 1 and 3, we obtain the HER under the alkaline conditions
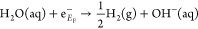
4Therefore, the absolute potential of the HER under the alkaline conditions is *E*_H_2_O/H_2__^°,abs^ = *E*_H^+^/H_2__^°,abs^ – 2.30*RT* × p*K*_w_. Taking a p*K*_w_ of 14 for neat water, one obtains an *E*_H_2_O/H_2__^°,abs^ of 3.62 V, and it becomes the supposed value of −0.83 V on the SHE scale.

Similar to eq 2, the corresponding HER potential under the alkaline conditions in concentrated electrolyte solutions and on the SHE scale can be expressed as
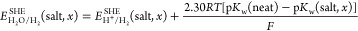
5

Inspecting eqs 2 and 5, one can see that the intrinsic HER activity is strongly influenced by how the proton work function and the acidity constant of water change with respect to the salt concentration. Fortunately, both quantities can be accessed from molecular modeling.

The computation of p*K*_a_ (and redox potentials) in aqueous solutions within the context of DFTMD has been worked out previously by Cheng, Sprik, and co-workers using the proton insertion method.^14,15^ It involves calculations of the deprotonation free energy of hydrated hydronium ions (proton), Δ_dp_*A*_H_3_O^+^_, and that of a hydrated water molecule, Δ_dp_*A*_H_2_O_, calculated via free energy perturbation theory. For the computational details, including the system setup and free energy calculation method, see the Supporting Information. These DFTMD simulations with the BLYP functional^16,17^ were carried out with CP2K code^18,19^ in LiCl solutions of concentrations from 0.0 to 9.3 *m*.

In contrast, the computation of *W*_H^+^_ is more involved. It entails a non-negligible electrostatic contribution, i.e., Poisson potential ϕ of the liquid system under periodic boundary conditions. Proton work function *W*_H^+^_ in concentrated electrolyte solutions can be expressed as
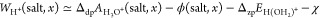
6where the first three terms on the right-hand side of the equation together equal the negative of the proton solvation free energy (−Δ_solv_*G*_H^+^_). Δ_zp_*E*_H(OH_2_)^+^_ is the zero-point (zp) energy of the H_2_O^+^–H bond, which was calculated to be ∼0.35 eV.^14^ χ is the surface dipole potential present at the liquid–air interface, which is a nonmeasurable quantity but estimated to be ∼170 meV.^20^ It is worth noting that surface dipole potential χ is much smaller than the Poisson potential of the liquid phase itself. We assume here that χ is a constant that is independent of salt concentration. Therefore, to determine *W*_H^+^_, one needs to know how the Poisson potential of the liquid phase shifts with the salt concentration in addition to deprotonation free energy Δ_dp_*A*_H_3_O^+^_.

To determine the Poisson potential of bulk electrolyte systems, we have explored the finite-field MD simulations^21−23^ of bulk electrolyte solutions to accelerate the statistical convergence. Because electrolyte solutions are ionic conductors, Maxwell electric field **E** vanishes on statistical average and electric displacement **D** is related to supercell polarization **P** by the relation **D** = 4π⟨**P**⟩. This allows us to apply classical MD simulations at *D*_*z*_ = 0 with the MetalWalls code^24,25^ to the same bulk electrolyte solution systems that were used in the DFTMD simulations (for the deprotonation free energy calculation). The extended simple point-charge (SPC/E) water model^26^ and Joung–Cheatham (JC) ion models^27^ were used in these additional classical MD simulations, which have been validated before in terms of both thermodynamic and transport properties at higher salt concentrations.^28,29^ Subsequently, the Poisson potentials were calculated with both DFT and point-charge models using a method summarized in Figure 2 and explained in the Supporting Information. The application of constant *D*_*z*_ = 0 MD simulations to resample the ionic configurations and a proper wrapping of ions are essential for ensuring a centrosymmetric profile of the resulting Poisson potential, i.e., ⟨*P*_*z*_⟩ = 0.

**Figure 2:**
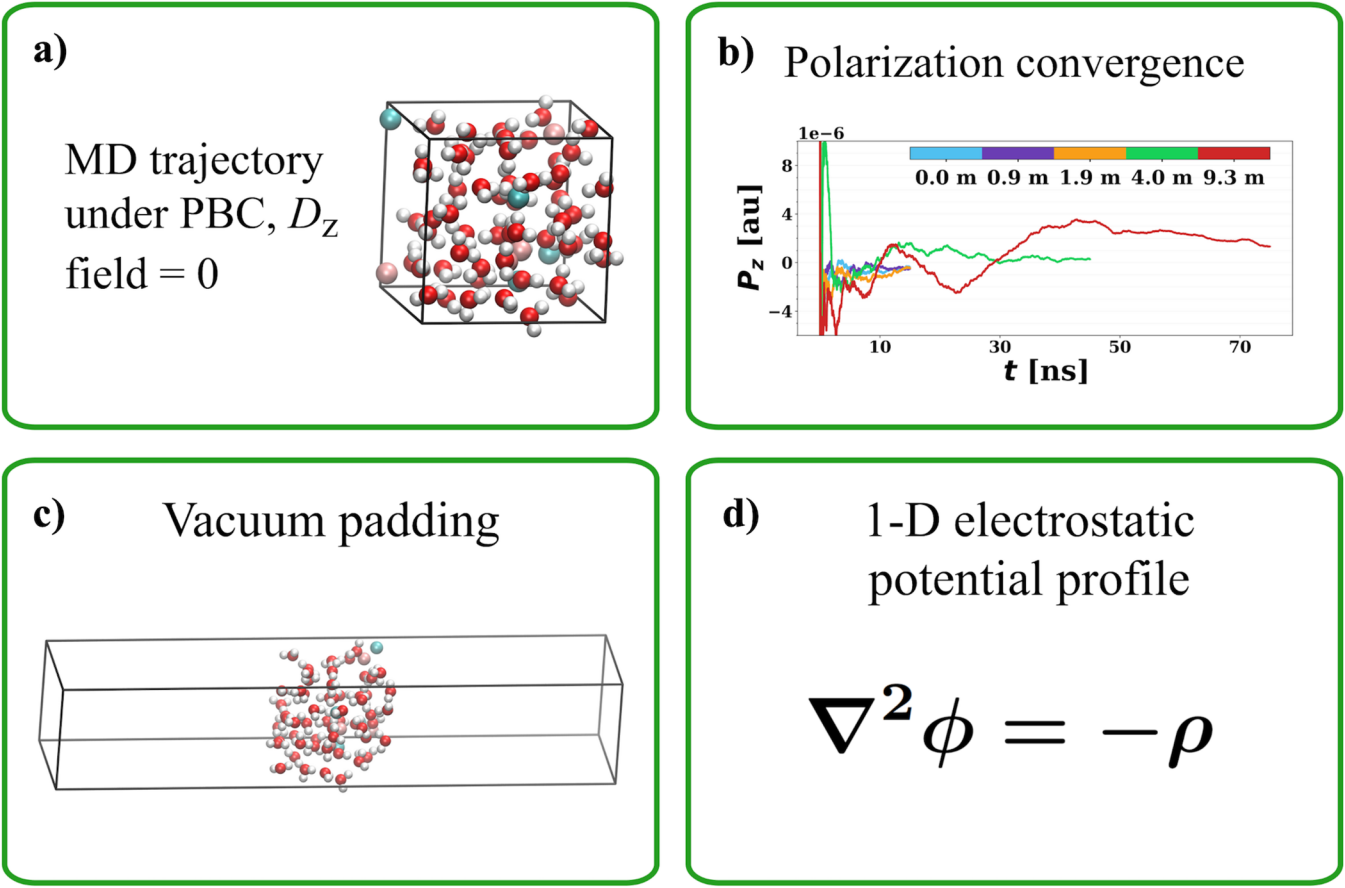
Scheme of the method for calculating Poisson potential profiles from simulation boxes of the LiCl electrolyte. In a) and b), a bulk simulation box of the electrolyte solution at the given concentration was simulated under constant *D*_*z*_ = 0 in the *z*-direction until supercell polarization *P*_*z*_ had converged. Then, in c), the simulation cell and the corresponding trajectory were extended to include a vacuum region. Finally, in d), they were used to determine the electrostatic potential in the bulk electrolyte by solving the Poisson equation for both DFT and point-charge models.

The main results from DFTMD simulations of the deprotonation free energy and Poisson potential as well as the proton work function and water dissociation constant are listed in Table 1. Both deprotonation free energy Δ_dp_*A*_H_3_O^+^_ and Poisson potential ϕ increase with salt concentration. However, the relative shift is larger in the case of the Poisson potential than the corresponding deprotonation free energy. As a result, proton work function *W*_H^+^_ decreases rather than increasing in concentrated electrolyte solutions. Therefore, the equilibrium potential of the HER under the acidic conditions becomes more positive. This confirm previous experimental observations of an increased proton concentration in concentrated electrolyte solutions.^7,8^ However, the situation is different for the HER activity under the alkaline condition. Water dissociation constant p*K*_w_ increases with salt concentration. Following eq 5, this positive shift in the water dissociation constant can counterbalance the negative shift in the proton work function. Similar ion enhancement effects on p*K*_a_ have also been observed for the silanol group.^30^ Interestingly, this thermodynamic balance appeared to be tipped to the side of a reduced HER activity, as recently reported with a similar computational methodology but for highly concentrated LiTFSI aqueous solutions.^10^ Therefore, our result suggests that one may tune the p*K*_a_ of buffer molecules to regulate the HER activity in concentrated electrolyte solutions.

**Table 1:** Computed Deprotonation Free Energies (Δ_dp_*A*_H_3_O^+^_), Poisson Potentials of the Liquid Phase (ϕ), Proton Work Functions (*W*_H^+^_), and Water Dissociation Constants (p*K*_w_) as a Function of the Salt Concentration in Aqueous LiCl Electrolyte Solutions^a^

LiCl (*m*)	Δ_dp_*A*_H_3_O^+^_ (eV)	*Fϕ* (eV)	*W*_H^+^_ (eV)	2.3*RT* × p*K*_w_ (eV)
0.0	15.30	3.49	11.29	0.82
0.9	15.35	3.53	11.30	0.78
1.9	15.41	3.61	11.28	0.73
4.0	15.46	3.74	11.20	0.90
9.3	15.67	4.03	11.12	0.93

a The standard work function of proton was determined to be 11.31(3) eV.^31^

Under either acidic or alkaline conditions, the Poisson potential contributes a major part to the shift in the HER potential in concentrated electrolyte solutions. Therefore, further discussion about its physical significance would be worthwhile. Many computational studies have been performed to determine single-ion solvation free energies and the closely related Poisson potential of the water–air interface.^14,32−34^ The water–air interfacial potential calculated with DFT takes on values of 3–4 V.^13,32,35^ In contrast, the water–air interfacial potentials of aqueous solutions calculated with classical point-charge models produce potentials 1 order of magnitude smaller with a negative sign.^32,36,37^ Furthermore, it has been established that the water–air interfacial potential cannot be constructed from molecular dipole moments alone but requires a significant contribution from the quadrupolar terms.^36−39^ Such a partitioning of the water–air interface potential was performed for DFT models in ref (35) by Leung. Using maximally localized Wannier functions, a partition into multipole moments was performed, showing that the quadrupole contribution to the surface potential amounts to 3.50 V, while the dipolar contribution is only 0.012 V. Later, Kathmann and co-workers^32^ presented an in-depth investigation of the potential landscape created by point-charge and density-based water models and showed how a sampling of the potential along a three-dimensional grid produces the characteristic inner potentials of the respective method. That is why in the literature, the Poisson potential of the liquid phase is also sometimes called the inner potential.^40^ Regardless of the different nomenclatures, a common conclusion from all of these studies is that the value (and the sign) of the Poisson potential of the liquid phase is strongly dependent on the nature of the underlying computational methods. From the Gibbs–Guggenheim principle,^41^ it is known that the difference between the inner potential can be measured but not the inner potential itself. Therefore, this raises the question of how the shift in this potential looks in concentrated solutions from computational models.

To answer this question, we have computed the Poisson potential of the liquid phase from both DFT and point-charge models for the same configurations of LiCl electrolyte solutions as a function of salt concentration. The results are shown in Figure 3. In the case of neat liquid water, we obtained a Poisson potential of 3.49 V with the DFT model (BLYP) and that of −0.86 V with the point-charge model (SPC/E water + JC ions). They agree very well with the literature values for both types of descriptions, 3.49 V^35^ and −0.84 V,^37^ respectively. It is worth noting that in our protocol of computing the Poisson potential of the liquid phase (Figure 2), we did not sample ionic configurations of any liquid–air interface. Therefore, the Poisson potential that we obtained does not contain the surface dipole contribution, and there is no double counting in eq 6.

**Figure 3:**
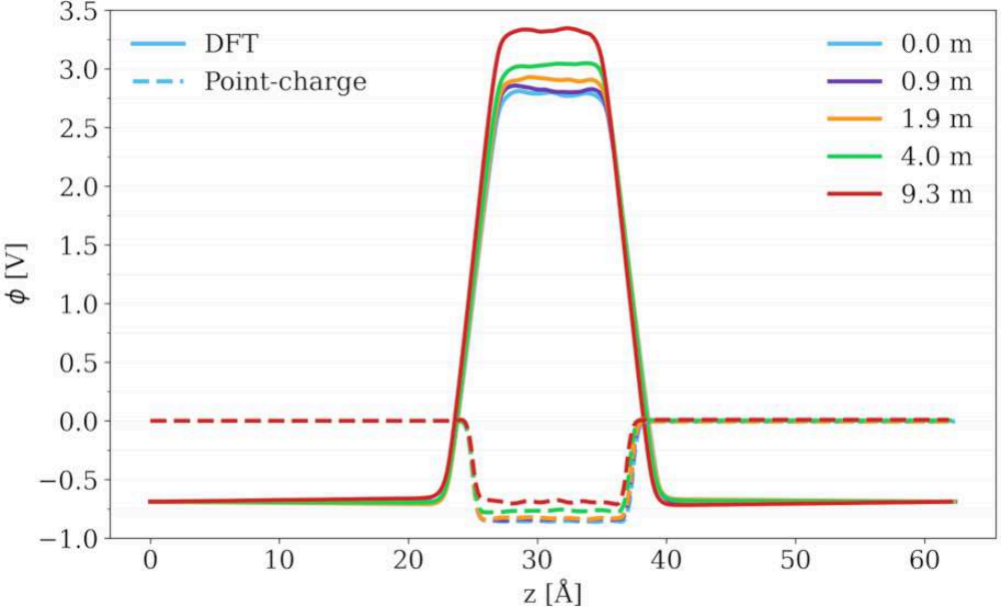
Poisson potential profiles as calculated from simulations of 0.0–9.3 *m* LiCl electrolytes. Dashed lines represent potentials calculated from a point-charge model (SPC/E water + JC ions), and solid lines represent potentials calculated from the DFT model (BLYP). The same trajectories of bulk electrolyte solutions generated with classical MD simulations at constant *D*_*z*_ = 0 have been used in both cases.

Unexpectedly, the shift in the Poisson potential of the liquid phase as calculated with a point-charge model (see Figure 4) has not only the same sign but also a comparable value compared to that of the DFT model, despite the fact that their absolute values are drastically different and even differ from each other by sign (see Figure 3). Thus, the factor contributing to this shift in the Poisson potential with the salt concentration must be physical. In fact, the values of our Poisson potential shifts are similar to that of the liquid Madelung potential as reported in ref (11), which is a positive shift of 0.35 V for the LiFSI/ethylene carbonate system going from 0.8 to 7.6 *m*. This does not come as a coincidence because these two potentials, the liquid Madelung potential and the Poisson potential of the liquid phase, are essentially the same physical quantities. Therefore, these results point out that the single-ion reactivity in concentrated electrolyte solutions undergoes a strong electrostatic modulation, and with that, it poses new questions. Shifts in activity due to the Poisson potential of the liquid phase are independent of the chemical identity and relate only to the net charge. Nevertheless, solvation free energies, which capture a fuller range of interactions, are not necessarily subject to the same concentration-dependent shift when compared across different but equally charged species. In the Li-ion battery research, Li^+^/Li rather than the SHE is used as the reference electrode. Given that the Li^+^/Li reference itself depends on the solvation free energy of Li^+^^42^ and the nature of the (organic) solvent,^43^ it would be rather interesting to see how the standard conversion factor, e.g., −3.04 V between the Li^+^/Li and the SHE, holds true in different types of concentration electrolyte solutions from both experimental and theoretical means. This will help to further clarify the thermodynamic basis of the reduced HER reactivity in WiSEs.

**Figure 4:**
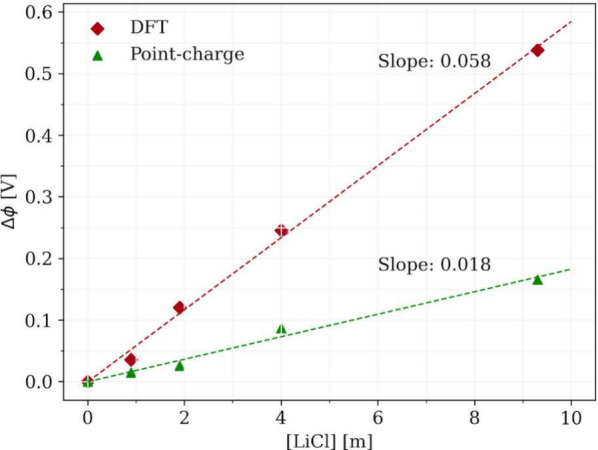
Poisson potential shifts for 0.0–9.3 *m* LiCl solutions as referenced to the value at 0.0 *m*. Red diamonds show the shifts calculated by the DFT model (BLYP), and green triangles show the same quantity calculated from the point-charge model (SPC/E water + JC ions).

## Supporting Information Available

Computational setups, calculations of the deprotonation free energy and the water p*K*_w_, electrostatic potential profile calculations, and error estimations in free energy calculation (PDF)
Transparent Peer Review report available (PDF)
