# Supplementary material for: Electrostatic Aspect of the Proton Reactivity in Concentrated Electrolyte Solutions
Source: J Phys Chem Lett. 2024 Dec 3;15(49):12212–7. doi: 10.1021/acs.jpclett.4c02923 (PMC11648079; doi:10.1021/acs.jpclett.4c02923)
Supplement: Supplementary file 1 — jz4c02923_si_001.pdf [file jz4c02923_si_001.pdf]

Supporting Information: Electrostatic aspect of the proton reactivity in concentrated electrolyte solutions

Alicia van Hees and Chao Zhang*

Department of Chemistry-Ångström Laboratory, Uppsala University, Lägerhyddsvägen 1, P. O. Box 538, 75121 Uppsala, Sweden

E-mail: chao.zhang@kemi.uu.se

Contents

1 **Computational setups** **S-3**

2 **Calculations of the deprotonation free energy and the water pK_w** **S-4**

3 **Electrostatic potential profile calculations** **S-5**

4 **Error estimations in free energy calculation** **S-7**

References **S-9**

1 Computational setups

Electrolyte simulation supercells were constructed by the modification of a 12.432 Å cubic box of 64 water molecules. Pairs of randomly selected water molecules were replaced with LiCl ion pairs, after which the box was scaled to match experimental density at 20 °C, taken from ref.[S1] Table S1 lists the composition (the number of ions and the number of water molecules) and the corresponding concentration in molality.

Table S1: Composition of the supercells used in this work.

LiCl [m]	No. of H_2O	No. of LiCl	Cell parameter [Å]
0.0	64	0	12.432
0.9	62	1	12.350
1.9	60	2	12.306
4.0	56	4	12.194
9.3	48	8	11.969

Classical molecular dynamics simulations, both the equilibration performed with the GROMACS code[S2,S3] and the constant displacement field simulations performed with MetalWalls, used the SPC/E point charge model of water. For Li^+ and Cl^- ions, the Joung-Cheatham III (JC-S)[S4] parameters were used. Systems with 0, 0.9, 1.9, 4.0, and 9.3 m LiCl were equilibrated in GROMACS for 1 ns with a time step of 2 ps at 300 K before DFTMD deprotonation free energy simulations were run.

All DFT calculations were carried out with the BLYP functional[S5,S6] and a TZV2P basis set, adapted to the Goedecker-Teter-Hutter (GTH)[S7 S8] pseudopotentials with the cutoff of 280 Ry. To counteract the known tendency of BLYP water to show slower than experimental dynamics,[S9] a temperature of 330 K was used instead. DFTMD simulations with a time step of 0.5 fs were performed in the CP2K/Quickstep software.[S10,S11]

2 Calculations of the deprotonation free energy and the water pK_w

Following the proton insertion method described in ref.,[S12,S13] the deprotonation free energy calculated $\Delta_\mathrm{dp}A_\mathrm{HA}$ from the Simpson numerical integral was used (Eq. 1) and $\Delta_\mathrm{dp}E_\mathrm{HA}$ is the vertial energy gap between the protonated and deprotonated states. These DFTMD simulations in 0, 0.9, 1.9, 4.0, and 9.3 m LiCl were run for 100 ps at each coupling parameter λ value.

$$\Delta_\mathrm{dp}A_\mathrm{HA} \approx \frac{\langle\Delta_\mathrm{dp}E_\mathrm{HA}\rangle_{\lambda=0} + 4\langle\Delta_\mathrm{dp}E_\mathrm{HA}\rangle_{\lambda=0.5} + \langle\Delta_\mathrm{dp}E_\mathrm{HA}\rangle_{\lambda=1}}{6} \tag{1}$$

.

Calculations of water pK_a describe the process where a proton is removed from a water molecule and transferred to its aqueous solvated state. The latter, being represented by a constrained hydronium ion, is not a perfect approximation of the solvated proton. According to ref.[S12] the largest correction is that of the proton translational entropy, and this is the only correction included in the present work. Thus, our p$K_\mathrm{a}$calculations were performed as follows:

$$\ln 10 k_B T \mathrm{p}K_a = \Delta_\mathrm{dp}A_\mathrm{HA} - \Delta_\mathrm{dp}A_{\mathrm{H_3O^+}} + k_\mathrm{B}T\ln\left[c^\circ \Lambda^3_{\mathrm{H^+}}\right] \tag{2}$$

The translational entropy correction term (last term on the right hand side) is composed of the solute standard concentration c° and the thermal wavelength of the proton. Its value is 0.19 eV or 3.2 p$K_\mathrm{a}$units. The simulations of water deprotonation free energy had a production time of 50 ps and a time step of 0.5 fs. For the calculations presented in this work, the deprotonation free energy of water and the (de)solvation free energy were calculated in separate simulations.

In the proton-insertion scheme, it was necessary to define a location where the proton

were to be inserted during the simulations where the other atoms were propagated by a Hamiltonian not including any interaction with said proton. To this end, a "dummy atom" (denoted d in this text) was used in place of the hydrogen nucleus when the latter was not present. This dummy atom was tied to its (randomly chosen) parent water molecule with target O-d bond lengths 1 Å and target H-d bond lengths 1.65 Å in order to ensure sensible structures upon proton insertion. These restraints were kept also for the force evaluations where the hydrogen atom interacted normally with its surroundings. Additionally, all water molecules were subject to O-H bond length restraints targeted at 1 Å, preventing "spectator" water molecules from partaking in proton transfer.

3 Electrostatic potential profile calculations

Electrostatic potential profiles were obtained from samples of classical MD simulations. Compositions remained the same as in the DFTMD free energy calculations, but classical MD with the MetalWalls code[S14,S15] at the displacement field $D_z = 0$ in the z-dimension was used to enhance the sampling and converge the electrostatic profile. In the interest of comparing DFT and point-charge models with respect to the Poisson potential shift, the same trajectory at each salt concentration was used to calculate two different sets of electrostatic potential profiles.

For every classical MD trajectory, 500 evenly distributed frames were used to calculate the potential profile using DFT and the same computational setup as in the free energy calculations, with the addition of a $D_z = 0$ constraint applied also to the solutions of the electronic density. In parallel, the point charges of the classical MD computational setup was used to calculate the potential profile using every 0.25 ps time step of the simulation. The length of the simulations are presented in table S2.

To find the electrostatic potential of the electrolyte solutions with respect to a common reference, the geometries from the snapshots were padded with 25 Å of vacuum in the z-

Table S2: Simulation lengths with the classical MD simulations at $D_z = 0$ to converge the polarization P_z.

LiCl [m]	Simulation length [ns]
0	15
0.9	15
1.9	15
4.0	45
9.3	75

direction, after which average potentials could be determined with respect to the vacuum level.

For the polarization of the simulation box to be continuous in time, the itinerant polarization must be considered when simulating the system with PBC. This means that a charged species "leaving" the central simulation box through a boundary in terms of polarization is considered to continue its movement outside of the box, even though from the perspective pf a single supercell it is wrapped to the other end. This is thus the value of the polarization that is minimised through application of a $D_z = 0$ field. When padding simulation systems with vacuum, geometrical wrapping is needed in order to conserve the bulk density of the slab. The itinerant polarization minimised during MD simulations does not directly translate to this setup. Therefore, a correction scheme was applied that only allowed for geometrical wrapping events that did not shift the polarization branch from the itinerant value. In practice, this meant that ions were not wrapped to the other end of the slab unless an ion of the opposite sign was wrapped the same way or an ion of the same sign was wrapped the opposite way, thus preventing a discontinuous jump in the polarization.

After the profile had been calculated, the average potential of the electrolyte phase was determined by fitting its rise with a tanh function:

$$a[\tanh(b(z + c)) + 1] \tag{3}$$

It should be noted that the averaging of potentials in time and space produced very flat z-dimensional potentials (see for example Figure 3 in the main text), meaning that the

computed Poisson potential was not very sensitive to the exact method of fitting.

Table S3: Computed Poisson potentials ϕ of 0.0-9.3 m LiCl using BLYP-DFT and SPC/E + JC-S models of the electrolyte solutions. The shift in the Poisson potential $\Delta\phi$ with respect to the neat water (0.0 m) are also included.

LiCl [m]	ϕ [V]		$\Delta\phi$ (V)	
	BLYP-DFT	SPC/E + JC-S	BLYP-DFT	SPC/E + JC-S
0.0	3.49	-0.86	0.00	0.00
0.9	3.53	-0.84	0.04	0.02
1.9	3.61	-0.83	0.12	0.03
4.0	3.74	-0.77	0.25	0.09
9.3	4.03	-0.69	0.54	0.17

4 Error estimations in free energy calculation

Error estimates were based on fitting the error estimates of block averages as described in ref.[S16] For a range of block lengths, the total simulation was split into sections each spanning that length of time. The standard error of the data set was calculated and the error of its mean was calculated by dividing by the square root of the number of elements in that set. If one gathers data for sufficiently much longer than the correlation time of fluctuations of the desired quantity (here the vertical energy gap), the standard errors of the block average will approach the true error and thus converge. The limiting value can be found by fitting the trend with one or more exponentials corresponding to different events with different characteristic times. By visual inspection, some block error series were best fitted with a single-exponential and some with a bi-exponential function (Fig. S1). For consistency, the error estimated from a bi-exponential fitting was used for all data, as the final estimates were generally larger using this functional form. This procedure has been applied to the error estimation of individual $\langle \Delta_{\mathrm{dp}} E_{\mathrm{HA}} \rangle_\lambda$ and the error propagation has been applied to estimate the final error for both the deprotonation free energy and the pK_{a}. These results are shown in Table S4.

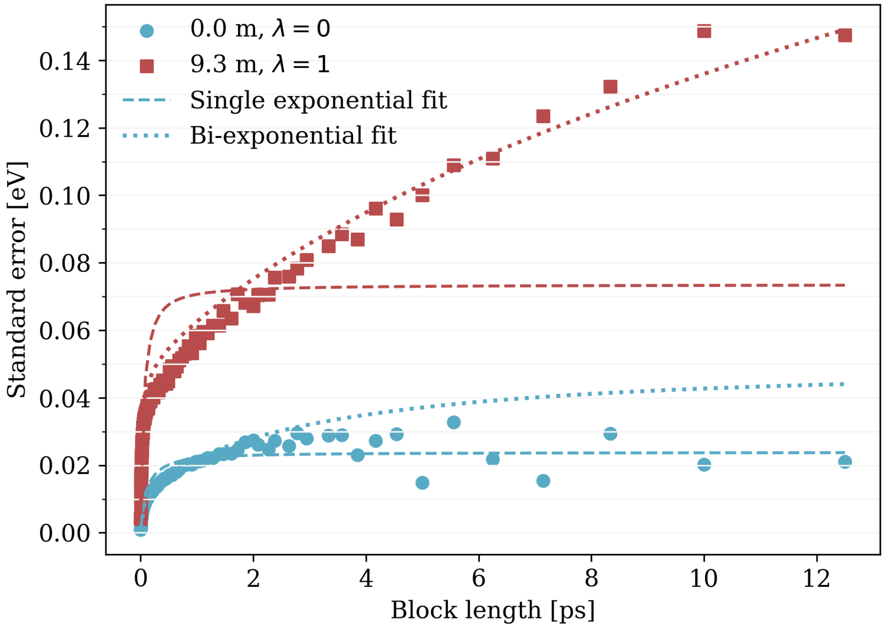

Figure S1: Block average errors in $\langle \Delta_{\mathrm{dp}} E_{\mathrm{H_2O}} \rangle_\lambda$ for two selected simulations. Discrete data points correspond to the standard error calculated from instantaneous values of $\langle \Delta_{\mathrm{dp}} E_{\mathrm{H_2O}} \rangle_\lambda$ divided into blocks along the trajectory. As shown in ref.[S16] the convergence of this value with block size gives an estimate of the error of the trajectory average. Both single- and bi-exponential fitting functions are plotted for both data series.

Table S4: The computed deprotonation free energy $\Delta_{\mathrm{dp}} A_{\mathrm{H_3O^+}}$, $\Delta_{\mathrm{dp}} A_{\mathrm{H_2O}}$ and the water pK_{w} as a function of the salt concentration in aqueous LiCl electrolyte solutions. The estimated errors are listed in the bracket next to the numbers.

LiCl [m]	$\Delta_{\mathrm{dp}} A_{\mathrm{H_3O^+}}$	$\Delta_{\mathrm{dp}} A_{\mathrm{H_2O}}$	pK_{w}
0.0	15.30 (2)	16.32 (2)	13.9 (3)
0.9	15.35 (3)	16.33 (3)	13.2 (4)
1.9	15.41 (3)	16.34 (4)	12.4 (4)
4.0	15.46 (5)	16.56 (3)	15.3 (5)
9.3	15.67 (5)	16.80 (7)	15.8 (8)

References

(S1) Wolf, A. *Aqueous Solutions and Body Fluids: Their Concentrative Properties and Conversion Tables*; Hoeber Medical Division, Harper & Row, 1966.

(S2) Berendsen, H. J.; van der Spoel, D.; van Drunen, R. GROMACS: A message-passing parallel molecular dynamics implementation. *Comput. Phys. Commun.* **1995**, *91*, 43–56.

(S3) Abraham, M. J.; Murtola, T.; Schulz, R.; Pall, S.; Smith, J. C.; Hess, B.; Lindahl, E. GROMACS: High performance molecular simulations through multi-level parallelism from laptops to supercomputers. *SoftwareX* **2015**, *1-2*, 19–25.

(S4) Joung, I. S.; Cheatham, T. E., 3rd Determination of alkali and halide monovalent ion parameters for use in explicitly solvated biomolecular simulations. *J. Phys. Chem. B* **2008**, *112*, 9020–9041.

(S5) Becke, A. D. Density-functional exchange-energy approximation with correct asymptotic behavior. *Phys. Rev. A* **1988**, *38*, 3098–3100.

(S6) Lee, C.; Yang, W.; Parr, R. Development of the Colle-Salvetti correlation-energy formula into a functional of the electron density. *Phys. Rev. B* **1988**, *37*, 785–789.

(S7) Goedecker, S.; Teter, M.; Hutter, J. Separable dual-space Gaussian pseudopotentials. *Phys. Rev. B* **1996**, *54*, 1703–1710.

(S8) Hartwigsen, C.; Goedecker, S.; Hutter, J. Relativistic separable dual-space Gaussian pseudopotentials from H to Rn. *Phys. Rev. B* **1998**, *58*, 3641–3662.

(S9) VandeVondele, J.; Mohamed, F.; Krack, M.; Hutter, J.; Sprik, M.; Parrinello, M. The influence of temperature and density functional models in ab initio molecular dynamics simulation of liquid water. *J. Chem. Phys.* **2005**, *122*, 14515–14515.

(S10) Kühne, T. D.; Iannuzzi, M.; Del Ben, M.; Rybkin, V. V.; Seewald, P.; Stein, F.; Laino, T.; Khaliullin, R. Z.; Schütt, O.; Schiffmann, F.; Golze, D.; Wilhelm, J.; Chulkov, S.; Bani-Hashemian, M. H.; Weber, V.; Borštnik, U.; Taillefumier, M.; Jakobovits, A. S.; Lazzaro, A.; Pabst, H.; Müller, T.; Schade, R.; Guidon, M.; Andermatt, S.; Holmberg, N.; Schenter, G. K.; Hehn, A.; Bussy, A.; Belleflamme, F.; Tabacchi, G.; Glöß, A.; Lass, M.; Bethune, I.; Mundy, C. J.; Plessl, C.; Watkins, M.; VandeVondele, J.; Krack, M.; Hutter, J. CP2K: An electronic structure and molecular dynamics software package - Quickstep: Efficient and accurate electronic structure calculations. *J. Chem. Phys.* **2020**, *152*, 194103–194103–47.

(S11) VandeVondele, J.; Krack, M.; Mohamed, F.; Parrinello, M.; Chassaing, T.; Hutter, J. Quickstep: Fast and accurate density functional calculations using a mixed Gaussian and plane waves approach. *Comput. Phys. Commun.* **2005**, *167*, 103–128.

(S12) Costanzo, F.; Sulpizi, M.; Valle, R. G. D.; Sprik, M. The oxidation of tyrosine and tryptophan studied by a molecular dynamics normal hydrogen electrode. *J. Chem. Phys.* **2011**, *134*.

(S13) Cheng, J.; Liu, X.; VandeVondele, J.; Sulpizi, M.; Sprik, M. Redox potentials and acidity constants from density functional theory based molecular dynamics. *Acc. Chem. Res.* **2014**, *47*, 3522–3529.

(S14) Marin-Laflèche, A.; Haefele, M.; Scalfi, L.; Coretti, A.; Dufils, T.; Jeanmairet, G.; Reed, S.; Serva, A.; Berthin, R.; Bacon, C.; Bonella, S.; Rotenberg, B.; Madden, P.; Salanne, M. MetalWalls: A classical molecular dynamics software dedicated to the simulation of electrochemical systems. *J. Open Source Softw.* **2020**, *5*, 2373–.

(S15) Coretti, A.; Bacon, C.; Berthin, R.; Serva, A.; Scalfi, L.; Chubak, I.; Goloviznina, K.; Haefele, M.; Marin-Laflèche, A.; Rotenberg, B.; Bonella, S.; Salanne, M. MetalWalls:

Simulating electrochemical interfaces between polarizable electrolytes and metallic electrodes. *J. Chem. Phys.* **2022**, *157*, 184801–184801.

(S16) Hess, B. Determining the shear viscosity of model liquids from molecular dynamics simulations. *J. Chem. Phys.* **2002**, *116*, 209–217.
