# Supplementary material for: Electrostatic Aspect of the Proton Reactivity in Concentrated Electrolyte Solutions
Source: J Phys Chem Lett. 2024 Dec 3;15(49):12212–7. doi: 10.1021/acs.jpclett.4c02923 (PMC11648079; doi:10.1021/acs.jpclett.4c02923)
Supplement: Supplementary file 2 — jz4c02923_si_002.pdf [file jz4c02923_si_002.pdf]

jz-2024-02923a.R1

Name: Peer Review Information for "Electrostatic aspect of the proton reactivity in concentrated electrolyte solutions"

First Round of Reviewer Comments

Reviewer: 1

Comments to the Author

The authors apply DFT-based MD, classical MD, and other theoretical techniques to investigate how the electrostatic affect the proton activity as the salt concentration varies in electrolytes. The paper is very well written, innovative, and is a very significant contribution to computational electrochemistry and battery model. I recommend publishing this work after minor but critical clarifications.

(1) LiCl is not quite LiTFSI or other salts typically used for water-in-salt electrolytes. The authors should acknowledge this point early on, and also justify the choice of LiCl.

(2) The authors seem to focus on the shift of H+ activity as the salt concentration varies relative to SHE. At least some of the experiments they discuss are however done in battery settings, typically with Li-containing anodes. For simplicity, let's choose Li metal (Li(s)) as anode. The two relevant half cell reactions are then

Li+ (in electrolyte) + e- -> Li(s) ; (A)

H2O (in electrolyte) + e- -> 1/2 H2 + OH- . (B)

So the salt concentration dependence of H2 evolution during true battery operation should also depend on the Li+ solvation free energies.

Recent work [Nature Energy 7, 1217 (2022)] on organic solvent batteries has shown the significant effect of changing solvent/salt on Li+ solvation free energies, and the "electrode potential," even at

~1.0 M salt concentration. In aqueous electrolytes, it is expected that high salt concentration will also significantly shift the potential associated with (A).

If the authors agree, please discuss how accounting for (A) will change their conclusion. If they don't agree please state the reason.

(3) I don't understand the last sentence of page 9/first line of page 10. Do the authors simply mean salt is not included in the "surface dipole potential" chi?

(4) "zp" in Eq. 6 presumably means zero-point; this should be explained.

(5) If the half cell Eq. (B) actually involves the reduction of a positively charged species, the "Madelung" effect would presumably cancel with that in (A). Is that true? This might be tested in experiments.

(6) Several references seem corrupted. Ref. 10 should be year 2023, and the volume/page numbers are missing. Ref. 11 is missing page numbers. The authors should proof-read the citations.

Author's Response to Peer Review Comments:

Dear Editor,

Thank you very much for the referee report and the corresponding decision letter dated November 11th, 2024. We are encouraged by the positive comments from the reviewer. In the following, please find our point-to-point response in the attachment. The requested non-scientific changes have been also implemented when applicable. We hope that the manuscript may be now accepted for publication in JPCLett.

Alicia van Hees and Chao Zhang

>> Please note that the marked version of the MS highlights the changes in red <<

Reviewer 1

The authors apply DFT-based MD, classical MD, and other theoretical techniques to investigate how the electrostatic affect the proton activity as the salt concentration varies in electrolytes. The paper is very well written, innovative, and is a very significant contribution to computational electrochemistry and battery model. I recommend publishing this work after minor but critical clarifications.

Response: We appreciate the very positive reviews of our work from the referee and the recommendation for publication.

(1) LiCl is not quite LiTFSI or other salts typically used for water-in-salt electrolytes. The authors should acknowledge this point early on, and also justify the choice of LiCl.

Response: We agree with the referee that LiCl is not quite the same as LiTFSI. Nevertheless, there are experimental investigations of HER activity in LiCl solutions, which serve as model systems for concentrated electrolyte solutions. In addition, it is more convenient from a computational point of view to work with a simple LiCl system rather than bulky anions such as TFSI-, which is another reason to start with the LiCl system in this study.

In the revised manuscript, we have acknowledged this point at the beginning of Page 3, Lines 1619 (the marked version).

(2) The authors seem to focus on the shift of H+ activity as the salt concentration varies relative to SHE. At least some of the experiments they discuss are however done in battery settings, typically with Li-containing anodes. For simplicity, let's choose Li metal (Li(s)) as anode. The two relevant half cell reactions are then

Li+ (in electrolyte) + e- ⟶ Li(s); (A)
H2O (in electrolyte) + e- ⟶ 1/2 H2 + OH- . (B)

So the salt concentration dependence of H2 evolution during true battery operation should also depend on the Li+ solvation free energies. Recent work [Nature Energy 7, 1217 (2022)] on organic solvent batteries has shown the significant effect of changing solvent/salt on Li+ solvation free energies, and the "electrode potential," even at ∼ 1.0 M salt concentration. In aqueous electrolytes, it is expected that high salt concentration will also significantly shift the potential associated with (A).

If the authors agree, please discuss how accounting for (A) will change their conclusion. If they don't agree please state the reason.

Response: We thank the referee for bringing up this interesting point. Agreeing with Nat. Energy paper brought up by the referee, we have also pointed out the important role of Li+ solvation free energies in determining the equilibrium potential in battery systems (*Mat. Today Energy*, **2022**, 28: 101060).

Indeed, we share the same opinion with the referee that the conversion between SHE and Li+/Li is no longer the textbook value of -3.04 V when increasing the salt concentration or changing the solvent. We have revised the manuscript to discuss this point together with point 5) on Page 11 and Lines 4-14 (the marked version).

(3) I don't understand the last sentence of page 9/first line of page 10. Do the authors simply mean salt is not included in the "surface dipole potential" chi?

Response: The sentence in question reads: "Therefore, the Poisson potential that we obtained does not contain the surface dipole contribution and there is no double counting in Eq. 6." Indeed, this requires some clarifications.

In our simulations, salt is always included except for the pure water system (m_{LiCl} =0). On the other hand, the experimental value of 170 meV of chi quoted in the manuscript was estimated for the water-air system without salt. That is why it is written in the manuscript, "We assume here chi is a constant that is independent of salt concentration". Nevertheless, there are experimental investigations that attempted to tackle this challenging and controversial issue, see for example, *Langmuir*, **2021**, 37: 7863.

(4) "zp" in Eq. 6 presumably means zero-point; this should be explained.

Response: We thank the referee for pointing this out. It has been corrected in the revised manuscript.

(5) If the half cell Eq. (B) actually involves the reduction of a positively charged species, the "Madelung" effect would presumably cancel with that in (A). Is that true? This might be tested in experiments.

Response: We agree the "Madelung" effect is insensitive to the nature of solvated ion. However, it does not mean the shift in the solvation free energies will be the same for Li+ and H+. Indeed, it would be rather interesting to investigate this point by both experimental and theoretical means. We have revised the manuscript to discuss this point together with point 2) on Page 11 and Lines 4-14 (the marked version).

(6) Several references seem corrupted. Ref. 10 should be year 2023, and the volume/page numbers are missing. Ref. 11 is missing page numbers. The authors should proof-read the citations.

Response: We thank the referee for carefully reading the reference section. The corrupted references have been corrected in the revised manuscript.

jz-2024-02923a.R2

Name: Peer Review Information for "Electrostatic aspect of the proton reactivity in concentrated electrolyte solutions"

Second Round of Reviewer Comments

Reviewer: 1

Comments to the Author

The authors have largely addressed my questions. I strongly recommend publishing this work in the Journal of Physical Chemistry Letters. It should become an important contribution to the recent high profile "potential shift" literature. I only have a few comments and optional suggestions.

1. There remain some typos/language issues, e.g., "solvtion" on page 6; "despite of" -> "despite on page 2.

2. The authors offer mathematical/computational explanation of the pKa and Poisson potential shifts. Can they offer a more physical rationale? It seems, as more Li+ are available, more water molecules are coordinated to Li+ and those water molecules should be more acidic. Is that

consistent with their simulation results?

3. The authors mention "Madelung" potential without defining
it. A definition (in the SI) would be welcome. A definition
of the Poisson potential would be useful too. They explain
it graphically and in words, but an equation (with constraints)
would help, and will provide contrast with "Madelung" -- if
there is any difference.

4. Wouldn't it be simpler and more intuitive to simulate an
electrolyte slab in vacuum instead of augmenting their bulk
electrolyte slab with vacuum, which may introduce artifacts?
A discussion would be welcome.

Author's Response to Peer Review Comments:

Dear Editor,

Thank you very much for handling our manuscript promptly!

Despite the very positive opinion from the referee, we are a bit surprised to see a completely new set of questions in the enclosed referee's report, which were not mentioned at all in the first round of the review. Nevertheless, we understand that the peer review process also functions as a means for scientific exchange and welcome that. Therefore, we have provided a detailed point-to-point reply letter in the attachment along with the typo-corrected manuscript. By choosing "transparent peer review", we hope the peer review files can be useful to the readers for observing these scientific discussions and serve as an extension to the paper itself.

As a long-term supporter of JPCLett, I hope that our manuscript may now be accepted for publication.

Sincerely yours,

Chao Zhang, on behalf of all authors

2024-11-23, Uppsala

Reviewer 1

The authors have largely addressed my questions. I strongly recommend publishing this work in the Journal of Physical Chemistry Letters. It should become an important contribution to the recent high-profile "potential shift" literature. I only have a few comments and optional suggestions.

Response: We thank the referee again for the very positive opinion and the recommendation for publication. It is slightly surprising to see that the referee raised new (computational) questions that did not show up in the first round of the review. We of course welcome them as healthy scientific exchanges in the peer-review process. On the other hand, we hope the referee understands it is not possible to resolve all the issues and accommodate all the discussions in a Letter.

1. There remain some typos/language issues, e.g., "solvtion" on page 6; "despite of" -> "despite on page 2.

Response: We thank the referee for pointing out the typos. They have been corrected in the revised manuscript.

2. The authors offer mathematical/computational explanation of the pKa and Poisson potential shifts. Can they offer a more physical rationale? It seems, as more Li+ are available, more water molecules are coordinated to Li+ and those water molecules should be more acidic. Is that consistent with their simulation results?

Response: We looked into the local coordination as a first reaction to interpret the results. However, there is no clear correlation between the local coordination environment and the properties that we investigated here, i.e. the Poisson potential shift and the pKa shift. It may not come as a total surprise since the Poisson potential is a global property and the pKa is also affected by it because the computation of pKa also uses the proton solvation free energy as the reference (see Eq. 2 in the Supporting Information).

3. *The authors mention "Madelung" potential without defining it. A definition (in the SI) would be welcome. A definition of the Poisson potential would be useful too. They explain it graphically and in words, but an equation (with constraints) would help, and will provide contrast with "Madelung" -- if there is any difference.*

Response: We refer the definition of "Madelung" potential to the work from Yamada and colleagues in Ref. 11. The definition of the Poisson potential is already given in the bottom right panel of Figure 2 by solving the Poisson equation from either DFT or point charge density, which is exact under the D=0 boundary condition. We will be rather happy to discuss the intricacy of these different potentials and the impact of electric boundary conditions on them when we get a chance to meet Yamada and his computational colleague face-to-face.

4. *Wouldn't it be simpler and more intuitive to simulate an electrolyte slab in vacuum instead of augmenting their bulk electrolyte slab with vacuum, which may introduce artifacts? A discussion would be welcome*.

Response: As explained to the referee in the first round regarding the question of the surface potential, it is a simplification rather than an artifact to not include the surface explicitly when computing the Poisson potential of liquid phase. We kindly refer the referee to the works from Cheng and Sprik (Ref. 14 and 15) for detailed discussions regarding this point, which also has a strong impact on the computations of the solvation free energy and the redox potential.
